# Cataclysmic Gastrointestinal Hemorrhage: Dreaded Complication of Metastatic Breast Cancer

**DOI:** 10.7759/cureus.25149

**Published:** 2022-05-19

**Authors:** Hanane Delsa, Sara Mounsif, Najwa Benslima, Mohamed Mahi, Fedoua Rouibaa

**Affiliations:** 1 Gastroenterology and Hepatology, Cheikh Khalifa International University Hospital, Mohammed VI University of Health Sciences (UM6SS), Casablanca, MAR; 2 Radiology, Cheikh Khalifa International University Hospital, Mohammed VI University of Health Sciences (UM6SS), Casablanca, MAR

**Keywords:** endoscopic variceal ligation, liver metastases, esophageal varices, upper gastrointestinal bleeding, breast cancer

## Abstract

Upper gastrointestinal bleeding (UGIB) from variceal rupture is a serious condition that can be life-threatening in some cases. Usually, the main cause is portal hypertension in cirrhosis, but other etiologies like liver metastases can be also involved. We present the case of a 64-year-old woman, with a history of metastatic breast cancer, who was admitted for a massive UGIB due to ruptured esophageal varices related to portal hypertension.

## Introduction

Acute bleeding from esophageal varices is a frequent and potentially life-threatening condition, associated with a six-week mortality rate of 10 to 20% [1] and a five-year mortality rate of more than 80% [2]. Its management must be urgent and must include initial stabilization, administration of vasoactive agents, endoscopic variceal ligation (EVL), and management of the underlying cause, generally acknowledged to be cirrhosis.

We describe a case of Upper gastrointestinal bleeding (UGIB) from variceal rupture in a patient with metastatic breast cancer and, through this case, we will underline the importance of searching for etiologies, other than cirrhosis, in case of bleeding esophageal varices.

## Case presentation

A 64-year-old woman was admitted to our emergency department for cataclysmic hematemesis and melena. She had a history of breast cancer treated with surgery (tumorectomy followed by a radical mastectomy, the reason was unclear) and chemotherapy nine years ago. The final histopathological study revealed a PT1c N1a M0 and grade 3 of Scarff-Bloom-Richardson (SBR) tumor, with negative surgical margins.

Recently, she suffered from diffuse abdominal pain leading to the discovery of liver and lung metastases of her breast cancer, Hormone Receptor (HR) positive and Human Epidermal growth factor Receptor (HER-2/neu) positive, confirmed by a liver biopsy. She was referred to her oncologist for palliative chemotherapy but she refused the treatment.

On admission, a physical examination found a conscious, stable patient with moderate mucocutaneous jaundice and bilateral lower limb edema. Vital signs were normal (pulse rate: 88 beats per min (pm), respiratory rate: 16 breaths pm, body temperature: 37.1°C, blood pressure: 100/55 mmHg). Abdominal clinical examination found subcutaneous collateral vessels, hepatomegaly, and shifting dullness. The rest of the physical examination was normal. The full laboratory workup revealed anemia with elevated liver enzymes. The results of the assessment are presented in Table 1.

**Table 1:** Laboratory workup results

	Value	Reference range
Hemoglobin	7.4 g/dL	11.5 - 17.5
Platelets	251 000/mm^3^	150 000 – 445 000
Prothrombin time (PT)	20.7 seconds	10-13
Prothrombin time ratio	50 %	70 - 100
Aspartate aminotransferase (AST)	501 U/l	< 35
Alanine aminotransferase (ALT)	110 U/l	< 35
Gamma-glutamyltransferase (GGT)	749 U/l	< 40
Total bilirubin	64.6 mg/l	< 12
Total protein	57 g/l	64 - 83
Sodium	129 mmol/l	136 - 145
Potassium	5.16 mmol/l	3.4 - 4.5
Cancer antigen 15-3	1 200 U/ml	< 26.2

**Figure 1:**
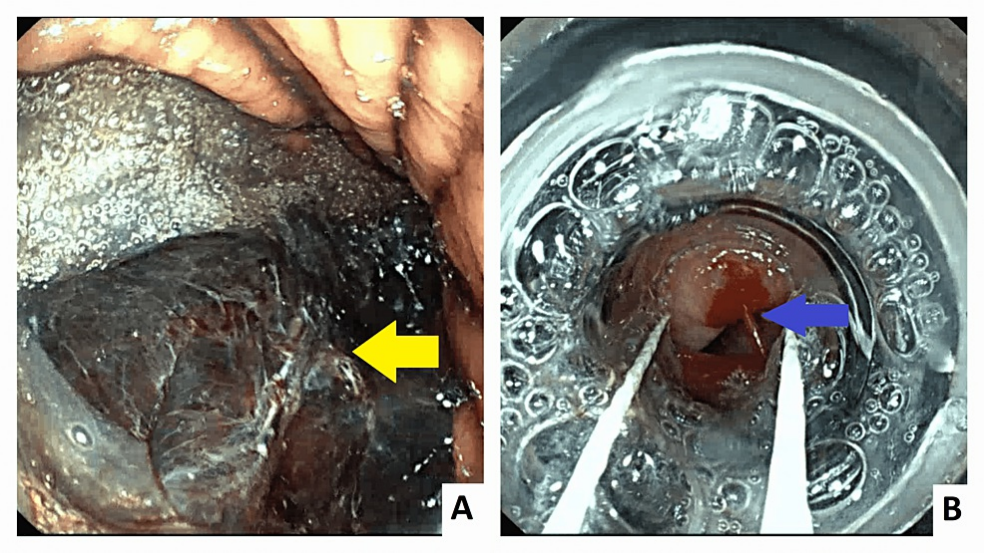
Esophagogastroduodenoscopy showed big blood clots in the stomach and esophageal varices A: big blood clots in the stomach (yellow arrow) B: grade 3 esophageal varices with active bleeding (Blue arrow)

**Figure 2:**
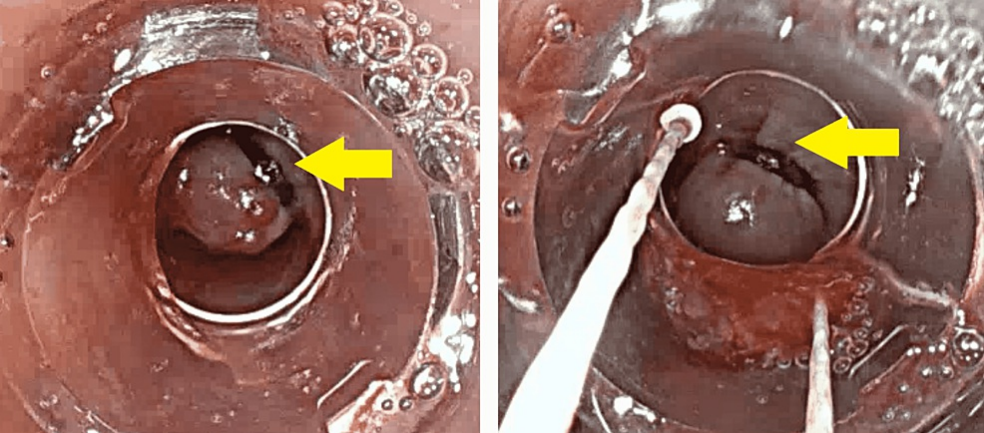
Endoscopic variceal ligation Yellow arrows in both right-hand side and left-hand side images show endoscopic variceal ligation

The patient was admitted to our intensive care unit and underwent a transfusion of two units of blood. Proton pump inhibitors and octreotide were administered intravenously. An esophagogastroduodenoscopy (EGD) was performed within six hours of her admission. It showed big blood clots in the stomach and grade 3 esophageal varices with red signs and active bleeding (Figure 1). Six bands were successfully placed without immediate complications (Figure 2).

**Figure 3:**
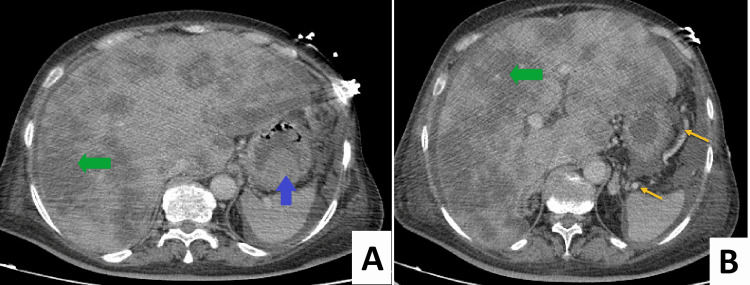
Axial CT scan Axial CT scan shows multiple liver metastases (green arrows in A and B), gastric blood (blue arrow in A), and perigastric collateral veins (yellow arrows in B)

The patient also received nonselective beta-blockers. The second session of endoscopic variceal ligation (EVL) was planned for 2 to 3 weeks. An abdominopelvic CT scan was also performed. It revealed multiple lung, bone, and liver metastases, and perigastric collateral veins (Figure 3) with abundant ascites.

The patient was referred to our oncology department for palliative chemotherapy. Unfortunately, one week later, she presented with an alteration of consciousness and another episode of hematemesis. A cerebral MRI was performed and ruled out the presence of brain metastases. The patient died within a few days.

## Discussion

Breast cancer is the most diagnosed cancer in women worldwide, with approximately 2.26 million cases recorded in 2020 [3]. It represents an important health problem with an increasing trend in prevalence and mortality rate [4]. Mortality in breast cancer increases in the metastatic setting, which occur in approximately half of breast cancer patients. The main sites of metastases are bone, liver, brain, and lungs.

Portal hypertension is an unusual complication of extensive liver metastases, however, few studies in the literature with only a small number of reported cases were published to report this association [5]. More recently, research has found that a lot of patients who received chemotherapy, and especially oxaliplatin, developed portal hypertension as part of the “porto-sinusoidal vascular disease” (PSVD). This term, proposed by the Vascular Liver Disease Interest Group, defines all the vascular liver diseases characterized by portal hypertension in absence of cirrhosis and other causes of liver disease [6].

The most dreaded complication of portal hypertension is upper gastrointestinal bleeding (UGIB) mainly caused by bleeding esophageal varices. It is an unusual manifestation of metastatic breast cancer but a life-threatening complication [7]. Bleeding esophageal, gastric, or colonic metastases; and acquired circulating anticoagulants are also reported [8]. A recurrent UGIB in metastatic breast cancer patients increases the risk of mortality and requires a specific treatment as well as repeated blood transfusions and hospital admissions. The management of acute variceal bleeding includes initial stabilization, appropriate medical treatment, and endoscopic intervention [7,9].

At first, patients should be appropriately resuscitated and transfused. Vasoactive agents (such as octreotide, somatostatin, or terlipressin), intravenous proton pump inhibitors (PPIs), and prophylactic antibiotics should be initiated as soon as possible [10]. After optimal resuscitation, EGD should be performed within 12 hours to evaluate the cause of hemorrhage and perform endoscopic therapy. In case of variceal hemorrhage, EVL must be performed to treat the active bleeding, and nonselective beta-blockers must be started [11].

The underlying disease-causing portal hypertension must also be treated to prevent a recurrence. In the case of liver metastases, various treatment options exist including chemotherapy, surgery, radiation, and targeted therapy. Despite these treatments, the prognosis is poor and the median survival after chemotherapy is 2-16 months [12].

## Conclusions

The occurrence of esophageal varices is a rare and unrecognized complication of breast cancer. It is mainly due to the presence of multiple liver metastases leading to portal hypertension. When ruptured, they can cause cataclysmic gastrointestinal bleeding, increasing the risk of mortality for those patients.
